# Gluten-free diet and quality of life in celiac disease

**Published:** 2014

**Authors:** Gabriel Samasca, Genel Sur, Iulia Lupan, Diana Deleanu

**Affiliations:** 1 Emergency Hospital for Children, Cluj-Napoca, Romania; 2 Department of Immunology, Cluj-Napoca, Romania; 3 Department of Pediatrics II, Iuliu Hatieganu University of Medicine and Pharmacy, Cluj-Napoca, Romania; 4 Department of Molecular Biology and Biotechnology, "Babes-Bolyai" University, Cluj-Napoca, Romania

**Keywords:** Celiac disease, Gluten-free diet, Quality of life

## Abstract

Many recent studies overshadow the effects of gluten-free diet. Gluten-free diet positive effects were observed in celiac disease patients: increase in body mass index, higher energy intakes, reducing adiposity gain, moderates the risk of the associated complications. However, adhering to a gluten-free diet is difficult for many people. A new solution is needed for quality of life of celiac disease patients, not for celiac disease treatment. Health education on gluten-free diet at home and in society seems to be the solution. The aim of our study is to evaluate the recent research on gluten-free diet as a nutritional therapy for patients with celiac disease. To achieve this purpose we have analyzed the published studies from 2008 to the present on nutrition in celiac disease.

## Introduction

Recent studies have shown that early diagnosis of celiac disease (CD) can improve quality of life (QOL) of these patients (1). From diagnosis to only therapy, which is gluten-free diet (GFD), CD is an interdisciplinary problem (2). Response to GFD is variable (3). What is the cause? Traces of gluten in GFD can produce damaging of the small intestine mucosa (4). For these reasons, recent research has focused on finding low or null toxicity wheat for CD patients. 

Our aim was to discuss the problems related to QOL and GFD. We conducted the study on PubMed database using keywords nutrition, CD. Eligibility criteria consisted of data found in PubMed database with reference to nutrition in CD from nutrition journals. 

**Dietary strategies started from the clinical effects of GFD**

Dietary strategies with regard to GFD and the research studies that analyze the gluten presence in wheat species occupy the largest share in the studied articles. Quality of life (QOL) of CD patients, the clinical effects of GFD, diagnostic problems in screening for gluten toxicity were also found in the studied articles. Our data shows that GFD positive effects were observed in CD patients: increase in body mass index, higher energy intakes, reducing adiposity gain, moderates the risk of the associated complications. Adhering to a GFD is difficult for many people.

On 149 CD children followed for 16 years, a significant (p=0.008) increase in body mass index (BMI) z-score after GFD was found (5). The frequency of overweight (12 % vs. 23.3 %, p=0.014) and underweight (16 % vs. 4.5 %, p<0.001) in children under GFD were substantially lower than that reported in tertiary care centers (6). A recent study concluded that 79 % from 413 patients (77 % were female and mean BMI was 24.1) with biopsy-proven CD had seen a dietitian (7). Martin et al. concluded that there was no significant association of either socio-economic deficiency or co-morbidities with adherence to GFD in CD patients (8). 

The researchers recognize long chain ω-3 fatty acids, plant flavonoids, and carotenoids as modulators for gene expression, oxidative stress and production of inflammatory mediators in CD (9). Therefore, adoption of dietary with these components could play a protective role against toxicity of gliadin peptides, keep intestinal barrier integrity and have a role in nutritional therapy of CD. Bergamo et al. evaluated the gliadin effects to DQ8 mice fed with a gliadin-containing diet with or without conjugated linoleic acid (CLA) supplementation and in differentiated human intestinal epithelial cell line (Caco-2). Gliadin was unable to generate oxidative stress and pathological consequences (10). Docosahexaenoic acid (DHA) counteracts many of the proinflammatory effect of arachidonic acid (AA). Vicentini et al. exposed a Caco-2 to gliadin peptides (PT-gl) (500 μg/ml) and DHA (2 μg/ml), both alone and simultaneously up to 24 h. The results showed that intestinal epithelial cells sustain the CD inflammation but cell culture studies have limitations (11).

A study revealed that CD patients are at the risk of having an inadequate intake of calcium, non-starch polysaccharides and vitamin D (12). Ohlund et al. aimed to assess the dietary intakes of energy and nutrients in children and adolescents on GFD. The conclusion was that children on GFD seem to follow the same trends as healthy children on a common diet, with high intakes of sucrose and saturated fat and low intakes of dietary fiber, vitamin D and magnesium (13). The dietary intake of CD children on a GFD and non-coeliac children were also evaluated. The observation was that CD children had higher energy intakes than controls, although BMI was comparable to the groups (14). Children and adolescents with CD are at risk for suboptimal bone health at time of diagnosis and after 1 year on GFD. This could be due in part to suboptimal vitamin D/K status. In conclusion, therapeutic dietary strategies for optimizing vitamin K/D intake are needed (15). Kautto et al. compared the energy and nutrient intakes among 13-year olds diagnosed with CD in early childhood with those of a non-coeliac (NC). The results revealed that both groups had low consumption of vitamin C, with 13% in the CD-group and 25% in the NC-group below estimated average requirements (EAR), and 21% of boys in the CD-group below EAR for thiamine (16). Therefore, some nutrient deficiencies need specific management (17). Many organizations were found for providing a dietary support and advice to the CD patients. Laparra et al. concluded that oral administration of Bifidobacterium longum ameliorates gliadin-mediated perturbations in liver iron deposition (18).

Other dietary proteins, such as those of cow's milk, were also investigated in CD. A study concluded that intolerance of CD patients to bovine milk is not due to CD + T cell stimulatory epitopes of gluten (19). Some other dietary proteins, such as alpha- and beta-caseins induce CD-like symptoms in some CD patients (20). Association between CD and primary lactase deficiency was also investigated. In CD patients, lactose intolerance could be owing to secondary lactase deficiency and to primary lactase deficiency but the hereditary lactase deficiency is frequent in CD children as in control population (21).

It is commonly accepted that breastfeed is a protective factor against CD (22). It is still not fully known whether breast-feed protects with permanent tolerance acquisition (23). At present, two schools declared that changing early feeding regimens in at-risk infants could either prevent the onset of the disease or merely delay it (24). Data on the impact on infant feeding are inconsistent (25). Soares et al. evaluated whether gluten exclusion can prevent adipose tissue expansion and its consequences. They feed the C57BL/6 mice with a high-fat diet containing 4.5% gluten or no gluten. Gluten-free animals presented a decrease in body weight gain and adiposity, without changes in calorie intake or lipid excretion. Their data suggested the beneficial effects of GFD in reducing adiposity gain, inflammation and insulin resistance. Therefore, GFD has been proposed as an option to the prevention of obesity (26).

**Analysis of the QOL of patients with CD**

Our data show that following a GFD is difficult for many people. GFD involves restriction of food choice (27). A group of researchers aimed to investigate the impact of CD and GFD on the lifestyle of patients and their families, together with recommendations for improvement of QOL. Their conclusion was that CD children have low compliance with the GFD: poor palatability (32 %), dining outside home (17 %), poor availability of products (11 %), and asymptomatic disease diagnosed by screening (11 %) (28). Better education about the disease, available gluten-free products, and proper food labelling could improve compliance and QOL (29). Unfortunately, adhering to a GFD is practically difficult. CD affects many of daily activities and gluten consumption is more common with possible consequences on health (30). CD patients have a diminished QOL in the social aspects of life. Women reported greater emotional responses to a GFD (31). About 90% from 98 adult patients with CD said they transgress the dietary pattern and about 58% consumed without knowing gluten products (32). The patients with non-celiac gluten sensitivity reported a slightly more difficulty in following GFD compare to CD patients (33). However, QOL studies suggest that CD patients benefit from a GFD. Furthermore, the GFD moderates the risk of the associated complications (34).

## Conclusions

A new solution is needed for QOL of CD patients, not for CD treatment. Health education on GFD at home and in society seems to be the solution.

## Acknowledgment

This paper was published under the frame of European Social Found, Human Resources Development Operational Programme 2007-2013, project no. POSDRU/159/1.5/S/138776.
